# From Fallopian Tube to Ovarian Cancer: Understanding the Evaluation and Management of Serous Tubal Intraepithelial Carcinoma Lesions

**DOI:** 10.1007/s11864-025-01346-0

**Published:** 2025-09-05

**Authors:** Vinita Popat, Ernest Han

**Affiliations:** Division of Gynecologic Oncology, Department of Surgery, City of Hope National Medical Center, Duarte, CA USA

**Keywords:** STIC, Ovarian cancer, High grade serous cancer, Peritoneal cancer, BRCA, Risk reducing salpingo-oophorectomy

## Abstract

Ovarian cancer, particularly high-grade serous carcinoma (HGSC), remains a leading cause of mortality in gynecologic oncology. Emerging research identifies serous tubal intraepithelial carcinoma (STIC) as a precursor lesion in many HGSC cases, highlighting its role in ovarian cancer pathogenesis and prevention. Management of STIC is challenging, as there is only limited data available to guide clinical decision-making. For average-risk women, opportunistic salpingectomy is increasingly being adopted during routine procedures such as hysterectomy or cesarean section. This intervention has demonstrated significant potential in reducing ovarian cancer incidence while maintaining safety and feasibility. For high-risk individuals, particularly BRCA mutation carriers, risk-reducing salpingo-oophorectomy (RRSO) remains the gold standard. RRSO significantly lowers ovarian cancer risk, though alternative approaches like salpingectomy alone or radical fimbriectomy are under investigation to preserve ovarian function in younger patients. To improve STIC detection, SEE-FIM pathology protocol is recommended when patients are undergoing risk-reducing surgery to prevent ovarian cancer, but challenges such as diagnostic variability and limited data persist. When STIC is detected incidentally, management varies based on risk factors and lesion characteristics. Genetic counseling and testing are essential when STIC is identified, as hereditary predisposition may guide further management. Surgical management is advised for cases of STIC with microinvasive carcinoma, but routine use of surgical management for STIC is not clearly defined in the literature. Bilateral oophorectomy is generally recommended when STIC is identified, and adnexal structures have not yet been removed. Chemotherapy is not recommended for treatment of STIC. Surveillance is suggested when STIC has been diagnosed, but there are no set guidelines as to the frequency and type of monitoring. Future directions include refining molecular profiling to predict progression and conducting randomized studies to establish evidence-based guidelines. Multidisciplinary collaboration is essential to optimize prevention and treatment, ultimately reducing HGSC incidence and improving patient outcomes.

## Introduction

Ovarian cancer remains one of the most formidable challenges in gynecologic oncology, characterized by significant morbidity and mortality. It has long stood as a leading cause of death among cancers arising from the female reproductive tract [1, 2]. Within this disease spectrum, high-grade serous carcinoma (HGSC) represents the most common histological subtype, demonstrating particularly aggressive behavior and poor survival outcomes [3]. Survival rates beyond the ten‐year mark have remained disappointingly low at around 30%, likely due to diagnosis at advanced stages [4]. This highlights the need for enhanced approaches in both prevention and early detection. Although screening tools for ovarian cancer have been explored extensively, widespread screening is generally not recommended due to inadequate specificity and sensitivity, as well as the lack of demonstrable mortality benefit [5, 6].

Given these challenges, a critical focus has been placed on identifying individuals at increased genetic risk, as targeted prevention strategies may offer the best opportunity to reduce ovarian cancer incidence. In recent years, there has been increasing recognition of genetic risk factors that predispose certain individuals to ovarian cancer. Individuals harboring germline mutations, such as BRCA1 and BRCA2, face a substantially increased lifetime risk of ovarian cancer as compared to the general population [7]. Consequently, many carriers of these pathogenic variants are counseled regarding the option of prophylactic surgery, typically in the form of risk-reducing salpingo‐oophorectomy (RRSO), to diminish the likelihood of future malignancy [8]. This preventive measure has proven effective in lowering the incidence of HGSC and thus remains central to the management strategy in high‐risk cohorts.

Understanding the pathogenesis of HGSC is essential for uncovering the mechanisms that drive cancer development and identifying key targets for early detection and prevention. Alongside advances in genetics, greater appreciation of the pathogenesis of HGSC has emerged. A wealth of evidence indicates that a notable proportion of these carcinomas originate in the fallopian tube, particularly at the tubal-peritoneal junction [9]. This area is believed to undergo significant epithelial transformation and, in many patients, is the apparent source of tumor precursor lesions that eventually seed the ovary or peritoneum [10, 11]. A spectrum of tumor precursor lesions has been identified along the tubal-peritoneal junction, including p53 signatures, serous tubal intraepithelial lesions (STILs), and serous tubal intraepithelial carcinomas (STICs), each representing a progressive step toward malignant transformation.

STICs are associated with as many as 60% of all HGSCs [12, 13]. With molecular evidence strongly supporting their role in the early stages of carcinogenesis, STICs have been suspected to be a direct precursor to HGSC. Given their potential role in the development of ovarian cancer, the detection and management of STICs may provide a crucial opportunity for cancer prevention. The recognition that STIC lesions can be significantly present, sometimes even in patients without hereditary risk, has generated interest in opportunistic salpingectomy for women who have completed childbearing. This proactive approach has the potential to reduce the incidence of ovarian and primary peritoneal malignancies later in life.

Though considerable progress has been made, many questions remain unanswered regarding STICs. In particular, if STIC represents a direct precursor to HGSC, a critical concern is determining the appropriate clinical course when these lesions are identified. Should the detection of STIC prompt immediate intervention, or is conservative management a viable option? Current data suggest that occult invasive carcinoma or peritoneal disease is detected in up to 30–60% of cases where STIC is found, raising concerns that some patients may already harbor microscopic cancer at the time of diagnosis [13]. This parallels the established progression model of endometrial intraepithelial neoplasia (EIN) as a precursor to endometrial carcinoma, where the presence of EIN often warrants definitive treatment with hysterectomy, due to its strong association with invasive disease [14]. However, unlike EIN, the natural history of STIC remains less well characterized, making management decisions more complex. Some clinicians advocate for routine surgical management and even chemotherapy in select high-risk scenarios, whereas others lean toward enhanced surveillance, highlighting the ambiguity stemming from the limited body of evidence [15] **. Achieving a uniform and standardized characterization of STIC—both morphologically and molecularly—is essential to ensure consistent diagnosis across pathology and clinical settings, reducing variability in interpretation and guiding more precise management strategies.

With continued research, the hope is that more personalized risk stratification and treatment algorithms will emerge, optimizing outcomes for patients diagnosed with these precursor lesions. This literature review will focus on the current understanding of STIC, its relationship with ovarian cancer and its management.

## STIC and Association with Ovarian Cancer

STIC is considered a rare entity in the general population, though growing awareness and the adoption of meticulous pathology protocols has likely increased its detection rate. Among patients diagnosed with HGSC, investigators have identified STICs more than half of the time during pathological review of the resected adnexa and in patients with STICs, concurrent HGSC is present 10.7% of the time [16, 17]. Patients with STICs identified on pathology may ultimately develop HGSC up to 29% of the time later in their life [18]. Historically, the incidence of STIC among those undergoing salpingectomy for benign indications has been reported to be under 1% [19]. However, in high-risk populations, specifically those with BRCA germline mutations who undergo risk‐reducing salpingo‐oophorectomy, STIC lesions have been noted in a substantially higher percentage of cases. Reports suggest that anywhere from 2 to 10% of BRCA‐positive individuals receiving RRSO might harbor STIC [16, 20]. Moreover, the presence of p53 signatures—an even earlier precancerous marker—can be found in as many as one‐quarter of these patients, further corroborating the notion that tubal involvement precedes ovarian involvement in the malignant process [20]. Risk‐reducing surgery offers a significant benefit in these high‐risk individuals, but even so, there remains a 0.4–0.9% residual chance of peritoneal carcinomatosis [18, 21]. This is possibly due to either undetected STICs, exfoliation of malignant tubal epithelium into the peritoneal cavity, or an alternative, non-tubal pathway of carcinogenesis. While the tubal hypothesis provides a compelling framework for understanding the origins of high-grade serous carcinoma, it may not fully account for all cases of ovarian and peritoneal malignancies [13]. Further research is needed to investigate non-tubal sources of carcinogenesis, which may contribute to the observed rates of subsequent peritoneal disease even after complete removal of the fallopian tubes and ovaries.

Besides genetic factors, there are other demographic and clinical variables that influence the incidence of STIC, further shaping individual risk profiles and guiding prevention strategies. Several demographic and clinical variables influence the incidence of STIC including age and germline pathogenic variant status. Notably, the likelihood of detecting STIC at the time of RRSO appears to increase with advancing age [18]. Another factor that has consistently emerged is the presence of multiple STIC lesions. In patients with high-risk pathogenic variants undergoing RRSO, more than 50% of patients with STICs had evidence of multiple tubal precursor lesions, suggesting that a higher number of precursor lesions may portend an increased risk of developing HGSC [18]. Overall, recognizing the interplay of these risk factors and identifying precursor lesions when present is a critical component of improving patient outcomes.

## How a STIC leads to Ovarian Cancer

Epithelial ovarian cancers have historically been described as malignancies arising from the surface epithelium of the ovary. Over time, however, this notion has shifted toward understanding the fallopian tube as the primary site of origin for many of these tumors, particularly the high-grade serous subtype [11]. The molecular events underlying malignant transformation in the adnexa involve an interplay between genomic instability and chronic exposure to oxidative stress, as well as other mediators of inflammation [22].

**Fig. 1:**
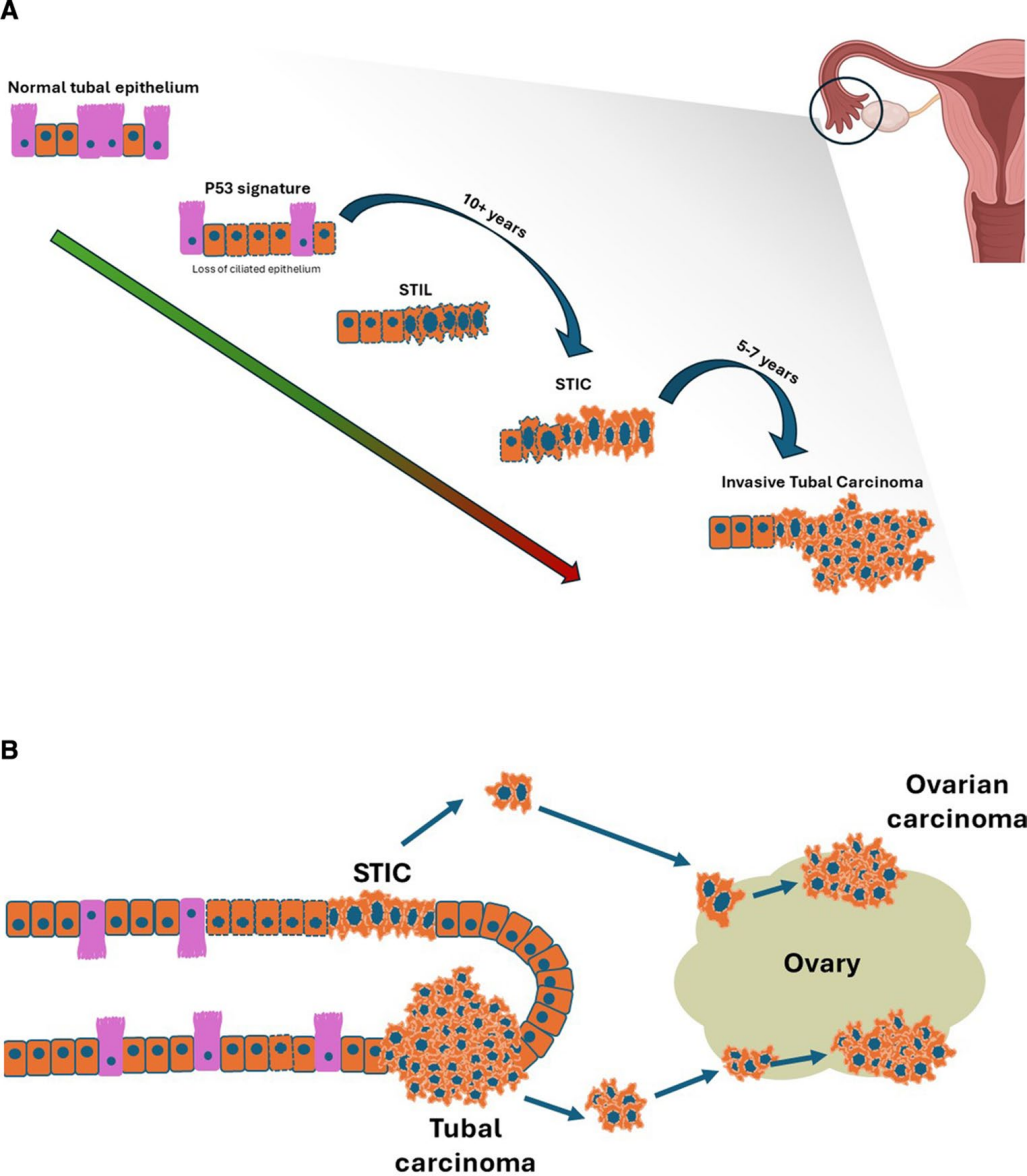
(**A**) Progression of normal tubal epithelium to development of ovarian HGSC (**B**) Proposed mechanism of shedding and implantation of either STIC or tumor cells from the tubal fimbriae onto the ovarian surface with subsequent development of ovarian cancer

Early molecular alterations are believed to manifest in the form of p53 mutations in otherwise normal-appearing fallopian tube epithelium [11]. Such changes can persist for years, possibly decades, before evolving into more advanced lesions [23]. Some of these early lesions are designated as “p53 signatures,” which can be present in normal tubal epithelia without overt histologic atypia [11]. Over the span of several decades, these p53 alterations may lead to greater genetic aberrations, culminating in STIC lesions. STICs, in turn, seem to have the capacity to progress in a relatively condensed timeline of 5–7 years into a frank HGSC (Fig. 1) [23].

While the tubal hypothesis provides a compelling explanation for the pathogenesis of many high-grade serous carcinomas (HGSCs), it does not appear to account for all cases of ovarian or peritoneal malignancies. Notably, not all ovarian or peritoneal cancers exhibit the classic p53 mutation-driven pathway, suggesting that alternative mechanisms of carcinogenesis must be present [11]. Similarly, not all STICs are associated with subsequent cancer development, and conversely, not all HGSCs have an identifiable STIC precursor. These observations raise important questions about the existence of non-tubal origins of ovarian cancer, which warrant further investigation.

One proposed alternative mechanism is the direct malignant transformation of residual Müllerian epithelial tissue, which includes the endometrium, cervix, and peritoneal inclusion cysts, as well as the potential contribution of endometriosis-associated malignancies [13]. Additionally, primary ovarian carcinogenesis remains a possibility, particularly in cases where precursor lesions are absent in the fallopian tube. While serous ovarian tumors are believed to frequently arise from tubal epithelium that has implanted on the ovarian surface, intrinsic ovarian epithelial transformation cannot be ruled out as a rare but distinct pathway [13].

The mechanical and environmental factors within the pelvic cavity also likely contribute to the pathogenesis of ovarian cancer. Repeated ovulatory events bathe the tubal fimbriae and adjacent peritoneum in follicular fluid, which contains elevated levels of reactive oxygen species, proteolytic enzymes, and other bioactive molecules [22]. These components can damage cellular DNA in the fallopian tube epithelium, especially at the fimbriated end. It is postulated that this microenvironment fosters the malignant transformation of the tubal mucosa, which eventually enables the spread of neoplastic cells to the ovary, omentum, and other peritoneal surfaces. There is a notion of “early metastasis”, that STICs can detach from the fallopian tube surface and migrate into the pelvis to implant on surrounding peritoneal tissue, ultimately manifesting as primary ovarian or peritoneal HGSC [23]. This provides a sound explanation for the occurrence of primary peritoneal HGSC in individuals who have previously undergone prophylactic removal of both ovaries, as undetected STICs may have already been seeded the peritoneal cavity before surgery. However, the persistence of peritoneal carcinomatosis in some individuals post-RRSO also suggests that alternative carcinogenic pathways may be at play, reinforcing the need for continued investigation into the full spectrum of ovarian and peritoneal cancer origins.

## Diagnosing STIC

The diagnosis of STIC largely depends on meticulous pathological examination of the fallopian tubes. There is currently no reliable imaging modality to detect these lesions preoperatively. In fact, STICs are often microscopic and asymptomatic, remaining elusive on standard ultrasound or cross-sectional imaging. Accordingly, diagnosis frequently arises as an incidental finding when surgical specimens from salpingectomy are subjected to detailed pathological review.

Morphologically, STIC is characterized by marked epithelial atypia, including cellular crowding, enlarged pleomorphic nuclei, and a loss of normal polarity [24]. Immunohistochemical analysis typically shows strong p53 overexpression (or complete absence in the case of a truncating mutation) and a high Ki-67 proliferative index [25]. This molecular profile helps to distinguish STIC from lower‐risk lesions such as STILs (serous tubal intraepithelial lesions), which may share some morphological changes but have a lower proliferation rate [24].

Pathologists now widely employ the Sectioning and Extensively Examining the FIMbriated end (SEE-FIM) protocol, a method that entails serial sectioning of the fimbrial portion of the fallopian tube at short intervals, thereby maximizing the likelihood of identifying subtle precursor lesions [26]. This approach is considered the gold standard in patients deemed high risk, particularly those carrying BRCA mutations or those undergoing RRSO for other hereditary syndromes [27]. Despite the recognized utility of SEE‐FIM, it has not yet been universally implemented among average‐risk patients in all clinical practices. Nevertheless, incidental STIC detection in low‐risk salpingectomies underscores the potential value of standardized protocols for pathologic evaluation. However, while universal adoption of SEE-FIM for all patients may enhance early detection, it presents a significant strain on resources, requiring considerable pathologist time, labor, and financial cost for identifying a relatively rare histologic entity—one that, even in high-risk cases, is still a precursor lesion rather than an overt malignancy.

While STICs most frequently arise in the tubal fimbriae, it is also important to recognize that they may appear elsewhere along the tubal lumen. Although rarer, involvement of the proximal tube has been documented, raising the possibility that any region of the salpinx harboring sufficiently damaged epithelium may become an incipient site of malignancy [28]. This highlights the importance of complete resection of the entire fallopian tube at time of resection and thorough pathologic examination to identify even small foci of neoplastic change.

It is important to note that the diagnosis of STIC can be subject to interobserver variability among pathologists, owing to the sometimes ambiguous histological distinction between early neoplastic changes and reactive epithelial atypia [29]. In a study conducted by Carlson et al., interobserver concordance in the diagnosis of STIC lesions was found to be at best fair to good, even among experienced gynecologic pathologists [29]. Notably, a proportion of STICs could not be consistently identified, highlighting the inherent subjectivity in pathologic interpretation. Given these challenges, corroboration of STIC diagnoses with a second observer may be warranted to enhance diagnostic accuracy and reduce variability [30].

Efforts are ongoing to improve diagnostic reproducibility through more precise criteria, standardized immunohistochemical (IHC) thresholds, and consensus guidelines [25]. In this context, Vang et al. have proposed an algorithmic approach to more uniformly identify STICs, stratifying cases into three morphologic categories: unequivocal STIC, suspicious for STIC, and not suspicious for STIC [24, 25]. To further refine classification, each category is then subcategorized using p53 and Ki-67 IHC staining, as STIC lesions are often p53-positive and demonstrate high Ki-67 proliferative indices. This method has been shown to increase diagnostic confidence and concordance among pathologists, but it has yet to be validated in larger studies. Nevertheless, the routine use of p53 and Ki-67 IHC on lesions suspicious for STIC may serve as an adjunctive tool to confirm diagnosis and enhance diagnostic uniformity [30].

Lastly, significant morphologic and molecular heterogeneity exists within lesions diagnosed as STICs, which may explain why some instances of STIC progress to malignancy, while others do not [31]. As further molecular characterization is undertaken, it is hoped that distinct subsets of STICs can be identified—those that are highly associated with progression to HGSC and predictive of poor outcomes, versus those that may remain indolent. This level of stratification could provide critical insights into which patients may benefit from more aggressive intervention versus conservative management, ultimately refining the clinical approach to STIC diagnosis and treatment.

## Management of STIC and Prevention of Ovarian Cancer

### Opportunistic Salpingectomy in the General Population

One of the most pivotal developments in ovarian cancer prevention in recent years has been the adoption of opportunistic salpingectomy in women who have completed childbearing. By removing the fallopian tubes during procedures such as hysterectomy or even at the time of cesarean section, it is believed that a significant proportion of future HGSCs could be prevented with an ovarian cancer risk reduction of approximately 80% [32, 33]. A landmark population-based retrospective cohort study in Canada further substantiated this association, revealing a significantly decreased incidence of ovarian cancer among patients who underwent opportunistic salpingectomy compared to those who did not [34]. This intervention has been shown to reduce up to 14.5% of ovarian cancer related deaths and decrease healthcare costs by million dollars annually [35]. Given the relative feasibility and safety of salpingectomy, major professional societies, including the American College of Obstetricians and Gynecologists (ACOG) and the Society of Gynecologic Oncology (SGO), now advocate for opportunistic salpingectomy as a routine prophylactic measure in average-risk women who have completed childbearing and do not desire future fertility [36–38].

Nonetheless, this strategy is not without caveats. A critical consideration is the possibility of preexisting, subclinical STIC lesions, which may have already disseminated into the peritoneal cavity by the time of surgery. In such cases, meticulous surgical technique—minimizing manipulation of the fallopian tubes and ensuring containment of the specimen—may reduce the risk of iatrogenic exfoliation of malignant or premalignant cells [15].While these precautions are theoretically sound, the empirical evidence supporting their efficacy remains limited and warrants further investigation.

Moreover, while opportunistic salpingectomy is generally safe—with no significant increases in postoperative complications, hospital readmissions, transfusion rates, or length of stay —clinical judgment must guide its application [39]. Specifically, the surgical approach should not be altered solely to facilitate salpingectomy, as the marginal benefit of the procedure does not justify additional morbidity associated with more invasive techniques [37]. As such, the implementation of opportunistic salpingectomy must be contextualized within broader surgical planning, patient characteristics, and ongoing research into optimal timing, technique, and long-term outcomes.

### Management in BRCA-Positive Patients

For carriers of BRCA1 or BRCA2 mutations, prophylactic removal of the ovaries and fallopian tubes is the standard-of‐care recommendation, especially after childbearing is complete. RRSO is recommended in patients with BRCA 1 at age 35–40 years and at 40–45 years for women with BRCA2, conferring a marked decrease in the risk of developing ovarian cancer and is associated with improved survival outcomes [5].

As demonstrated in the US Nurse’s study, premenopausal oophorectomy has been associated with significant detrimental impact on all-cause mortality [40]. Many women have delayed RRSO due to concerns for cognitive decline, increased risk for cardiovascular disease, osteopenia, and sexual dysfunction, symptoms associated with surgical menopause from oophorectomy [41, 42]. A meta-analysis found that 30–70% of high-risk women postpone RRSO, highlighting the need for alternative risk-reduction strategies and improved post-surgical management [42]. Bilateral salpingectomy alone may demonstrate a benefit for ovarian cancer risk reduction which is currently being studied in clinical trials [11]. An emerging concept known as “radical fimbriectomy” has been introduced in France as an alternative strategy for those who are reluctant to undergo oophorectomy at a younger age [43]. By removing the entire fallopian tube, including its fimbrial extremity and any adjoining ovarian tissue at the tubal–ovarian junction, this procedure aims to mitigate cancer risk while preserving ovarian endocrine function until natural menopause. Early results have been encouraging, with some studies reporting zero incidence of HGSC over years of follow-up [43]. Nevertheless, ongoing trials, such as the SoROCk trial, comparing salpingectomy alone to the established gold standard of bilateral salpingo‐oophorectomy, will be instrumental in determining whether delaying oophorectomy is safe and effective in high‐risk populations [44]. We caution that although salpingectomy alone might reduce risk, it has not yet been proven to match the effectiveness of removing both tubes and ovaries.

Given the recognized association between STIC and hereditary predisposition, any patient diagnosed with an incidental STIC and unknown mutation status should be offered thorough genetic counseling and testing [27, 45]. In certain studies, up to 12% of previously untested individuals discovered to have STIC on a surgical specimen were subsequently found to carry a BRCA pathogenic variant or an unclassified variant [46].

Additionally, the role of routine peritoneal biopsies in the setting of RRSO has been a subject of debate. While some clinicians may seek to identify occult disease, others point out that random peritoneal biopsies sample only an infinitesimal fraction of the peritoneal cavity and rarely alter clinical management [47, 48]. Moreover, such sampling may add operative time, cost, and potential morbidity. Similarly, the role of pelvic washings is also often controversial with mixed practice patterns based on provider and institution, as some providers routinely obtain pelvic washings and some never obtain washings. This is likely due to the relatively rare incidence of positive pelvic washings and limited data regarding clinical management in the setting of positive washings [49, 50]. The potential identification of isolated positive pelvic washings or in conjunction with STIC may suggest microinvasive spread of cancerous or precancerous lesions that may have additional clinical implications and influence management decisions [51]. In the United States, it is routine practice to obtain pelvic washings during RRSO. If a STIC is identified with positive washings, management is usually discussed at a multidisciplinary gynecologic oncology tumor board following complete staging.

## Recommendations for Management of STIC Lesions

When a STIC is incidentally identified, management options fall into several main categories: (1) further surgical treatment, including completion staging; (2) chemotherapy; and (3) surveillance. Determining the most appropriate course of action is rarely straightforward, largely because of the rarity of isolated STIC and the paucity of randomized data.

### Surgical Management

Rarely, STICs may be identified with an incidental microscopically invasive HGSC component that is not grossly visible at time of RRSO. If a STIC appears to be associated with an occult invasive HGSC, standard protocols dictate that such a case be managed as an outright malignancy [27]. In this scenario, treatment would typically involve total abdominal hysterectomy, bilateral salpingo-oophorectomy, omentectomy, and lymph node assessment, following guidelines for early‐stage ovarian cancer. Beyond the question of microinvasion, ‘staging’ surgery is not consistently performed for STIC, and prognostic implications are unclear. Multiple studies have demonstrated a reduction in cancer recurrence after surgical management, however the sample sizes of such studies are too small to conclude definite clinical benefit [52, 53]. Additionally, the definition of ‘staging’ surgery appears to vary amongst providers, defined as a combination of bilateral salpingo-oophorectomy, hysterectomy, omentectomy, and lymphadenectomy. The latest ESMO-ESGO-ESP consensus guidelines recommend complete surgical staging in the setting of incidental STIC. This was based on a meta-analysis study from Steenbeek et al. where the estimated hazard ratio was 33.9 (95% CI, 15.6–73.9) to develop peritoneal carcinomatosis during follow up in BRCA mutation positive women who underwent RRSO and was found to have STIC [18]. The risk of peritoneal carcinomatosis increased over time up to 27.5% after 10 years. The risk was only 0.9% for women without STIC at RRSO. The ESMO-ESGO-ESP group recommend staging of the peritoneum, preferably by minimally invasive procedure [27]. The group also recommends consideration of hysterectomy for patients with germline BRCA1 mutation or endometrial sampling if uterus is preserved. Lymphadenectomy was not recommended by the group. It is noteworthy that the supporting study by Steenbeek et al. was focused on BRCA mutated women undergoing RRSO. It is unclear if higher risk of peritoneal carcinomatosis also applies to BRCA wild-type women with STIC. Although staging is recommended, there is limited data to prove benefit to all STIC patients and thus it is important to consider risk factors for progression of malignancy including number of STICs (isolated vs. multiple), positive pelvic washing cytology, pathogenic genetic variant status, age, and desire for future fertility. In general, we recommend at minimum BSO in patients with an isolated STIC to identify other potential sites of malignancy, with the addition of hysterectomy based on risk factors and patient wishes.

### Chemotherapy

Adjuvant chemotherapy for isolated STIC is rarely recommended in the absence of invasive disease. The limited available data suggest that while STIC represents a bona fide precursor to HGSC, it may not necessarily progress to full-blown malignancy in every case, and systemic therapy carries risks of toxicity and deleterious effects on quality of life. Reports do exist of clinicians giving chemotherapy for STIC, particularly in cases with high‐risk features such as positive peritoneal cytology, but these situations are uncommon [18]. The consensus is that further research is required to ascertain whether there is any subgroup of STIC patients who genuinely benefit from adjuvant chemotherapy [15]. Until such evidence emerges, most guidelines counsel watchful waiting and reserve chemotherapy for individuals displaying more advanced pathology or any subsequent sign of disease progression.

### Surveillance

In many instances, surveillance is the most common recommendation for an incidental, isolated STIC in a patient who has already undergone bilateral salpingo-oophorectomy [15]. Surveillance strategies may include periodic pelvic imaging (ultrasound, MRI, or CT scans) and tumor marker assessment (e.g., CA‐125) [53–55]. However, it is well acknowledged that these screening modalities did not prove efficacious in the general population for early detection of ovarian cancer [6]. Their utility may nevertheless be comparatively more favorable in a high‐risk or STIC‐positive population, although the exact surveillance interval and modalities are not standardized. As an additional consideration, clinicians must recognize that the latency period from STIC to HGSC can stretch across multiple years, underscoring the importance of long‐term follow‐up up especially based on meta-analysis from Steenbeek et al. [18].

Thus, it appears prudent to offer a risk-based approach. For women identified with STIC who also carry pathogenic variants in genes such as BRCA1 or BRCA2, the threshold to intervene more aggressively might be lower, especially if the woman is at an age or stage of life where completing prophylactic hysterectomy and oophorectomy is acceptable. Conversely, for a patient found to have STIC by chance who is otherwise low risk, is younger, or declines any further surgery, vigilant monitoring can be a defensible alternative, with acknowledgment of the uncertainty in the data.

## Conclusion

Serous tubal intraepithelial carcinoma stands at the intersection of emerging research in ovarian cancer pathogenesis and ongoing efforts to refine preventive strategies. With the mounting evidence that high-grade serous cancers frequently originate in the fallopian tube, STIC has become a focal point of interest, illuminating a stepwise process of malignant transformation that may unfold across many years [10, 11]. The increased incidence of STIC among patients undergoing risk‐reducing surgery for hereditary cancer syndromes further highlights its critical role in the carcinogenic sequence.

Despite this progress, key challenges persist. Standards for diagnosing STIC vary among pathologists, with interobserver variability often complicating clinical decision-making. On top of that, debates regarding optimal management strategies remain. Some providers favor comprehensive staging in all cases, while others adopt a more measured approach—choosing surgical interventions or adjuvant therapies based on specific risk factors. The absence of large, controlled studies on STIC has necessitated reliance on smaller retrospective cohorts, expert consensus, and the extrapolation of outcomes from more advanced diseases.

Nevertheless, a few overarching points have emerged. Complete surgical staging is generally advised for lesions that show any evidence of microinvasion, and bilateral salpingo-oophorectomy remains the benchmark of prophylaxis for women with substantial genetic risks. Patients with BRCA mutation who undergo RRSO and are found to have STIC may be at increased risk for peritoneal carcinomatosis. Surgical staging and close monitoring at least 10 years should be considered. Chemotherapy is seldom used in the absence of invasive disease, as no robust data currently demonstrate a meaningful survival benefit for patients with isolated STIC. Surveillance is thus a mainstay for many, though the parameters of that follow‐up are far from standardized.

Going forward, the field would benefit greatly from more robust molecular characterization of STIC lesions. Elucidating the genetic and epigenetic signatures that define which STICs are most likely to progress to invasive disease could open the door to targeted approaches for prevention or therapy [9]. Similarly, randomized prospective studies would be invaluable to compare clinical outcomes for varying management algorithms, providing a foundation for more definitive guidelines. The ultimate objective is to reduce morbidity and mortality from a malignancy that has long been detected at advanced stages and associated with dismal prognoses. Through integrating genetic testing, histopathologic expertise, and thoughtful clinical management, there is optimism that outcomes will improve for women at risk and that the next generation of standards will more definitively address the complexities of STIC and ovarian carcinogenesis.

In summary, STIC represents a significant insight into the pathogenesis of high-grade serous carcinoma, offering a potential window for intervention before malignant cells have disseminated. Yet, many facets of its clinical management remain unsettled. As clinicians, pathologists, and researchers collaborate across disciplines, it is hoped that future guidance will better delineate risk stratification, integrate patient preferences, and optimize both prevention and treatment. Until then, management decisions will likely continue to rely on expert consensus, individual patient factors, and close monitoring.

## Key References

- Negri S, Fisch C, de Hullu JA, van Bommel M, Simons M, Bogaerts J, et al. Diagnosis and management of isolated serous tubal intraepithelial carcinoma: A qualitative focus group study. BJOG. 2024;131(13):1851-61. 10.1111/1471-0528.17919.
  - This reference is of outstanding importance as it is a recent focus group analysis looking at currently available literature and the consensus opinion of 49 providers from 12 different countries discussing the management of STICs. This reference also reviews the standard of care practice patterns for management of STIC lesions across multiple countries and organisations.
- Ledermann JA, Matias-Guiu X, Amant F, Concin N, Davidson B, Fotopoulou C, et al. ESGO-ESMO-ESP consensus conference recommendations on ovarian cancer: pathology and molecular biology and early, advanced and recurrent disease. Ann Oncol. 2024;35(3):248-66. 10.1016/j.annonc.2023.11.015.
  - This reference is of importance as it provides the most defined set of guidelines for evaluation and management of STIC lesions produced by a professional organization.

## Data Availability

No datasets were generated or analysed during the current study.
